# Characterization of the L-Lactate Dehydrogenase from *Aggregatibacter actinomycetemcomitans*

**DOI:** 10.1371/journal.pone.0007864

**Published:** 2009-11-17

**Authors:** Stacie A. Brown, Marvin Whiteley

**Affiliations:** Section of Molecular Genetics and Microbiology, The University of Texas at Austin, Austin, Texas, United States of America; Auburn University, United States of America

## Abstract

*Aggregatibacter actinomycetemcomitans* is a Gram-negative opportunistic pathogen and the proposed causative agent of localized aggressive periodontitis. *A. actinomycetemcomitans* is found exclusively in the mammalian oral cavity in the space between the gums and the teeth known as the gingival crevice. Many bacterial species reside in this environment where competition for carbon is high. *A. actinomycetemcomitans* utilizes a unique carbon resource partitioning system whereby the presence of L-lactate inhibits uptake of glucose, thus allowing preferential catabolism of L-lactate. Although the mechanism for this process is not fully elucidated, we previously demonstrated that high levels of intracellular pyruvate are critical for L-lactate preference. As the first step in L-lactate catabolism is conversion of L-lactate to pyruvate by lactate dehydrogenase, we proposed a model in which the *A. actinomycetemcomitans* L-lactate dehydrogenase, unlike homologous enzymes, is not feedback inhibited by pyruvate. This lack of feedback inhibition allows intracellular pyruvate to rise to levels sufficient to inhibit glucose uptake in other bacteria. In the present study, the *A. actinomycetemcomitans* L-lactate dehydrogenase was purified and shown to convert L-lactate, but not D-lactate, to pyruvate with a K_m_ of approximately 150 µM. Inhibition studies reveal that pyruvate is a poor inhibitor of L-lactate dehydrogenase activity, providing mechanistic insight into L-lactate preference in *A. actinomycetemcomitans*.

## Introduction

*Aggregatibacter actinomycetemcomitans* is a Gram-negative, nonmotile, opportunistic pathogen that resides exclusively in the mammalian oral cavity [1] and has been proposed to be the primary cause of the tooth and gum disease known as localized aggressive periodontitis [2], [3]. Within the oral cavity, *A. actinomycetemcomitans* resides in the gingival crevice, defined as the microaerophilic region bounded by the tooth surface and the epithelium lining the gingiva. The gingival crevice is bathed in gingival crevicular fluid (GCF) which provides nutrients for a robust and complex community of microorganisms. As a serum exudate, GCF likely contains several potential carbon sources to support this microbial community, including glucose, lactate, and fructose [4], [5]. Competition for resources is high in this environment and the rate of consumption of carbohydrates is extremely rapid [6], [7], [8], likely due to the large number of oral streptococci.

**Figure 1:**
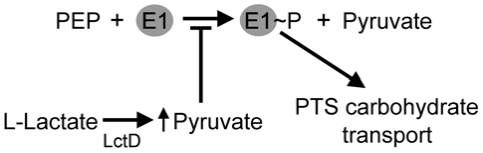
PTS substrate exclusion model. Lactate enters the cell through the lactate permease (LctP) and is converted to pyruvate by L-lactate dehydrogenase (LctD). Intracellular levels of pyruvate increase and prevent autophosphorylation of protein EI, thus inhibiting PTS-mediated carbohydrate transport. PEP is phosphoenolpyruvate.

We recently showed that although *A. actinomycetemcomitans* divides faster and achieves higher cell yields when catabolizing glucose, L-lactate is preferentially utilized [9]. Interestingly, L-lactate addition to a chemically defined medium inhibited *A. actinomycetemcomitans* uptake of glucose, a process referred to as PTS substrate exclusion [9]. Glucose transport in *A. actinomycetemcomitans* utilizes the phosphotransferase system (PTS). The PTS involves transport of glucose across the cytoplasmic membrane through a sugar-specific channel and concomitant phosphorylation upon entry into the cell to produce glucose-6-phosphate. The phosphoryl group originates from the phosphodonor, phosphoenolpyruvate (PEP), and is subsequently passed through a series of PTS proteins and ultimately to glucose (Fig. 1). The first step in PTS transport involves protein EI, which undergoes autophosphorylation in the presence of PEP to yield pyruvate and EI∼P. The phosphoryl group is then transferred to HPr, followed by a sugar-specific EII protein, which then phosphorylates the incoming sugar [10]. The intracellular ratio of PEP:pyruvate plays a crucial role in PTS transport. Indeed, as the PEP:pyruvate ratio declines, the model Gram-negative organism *Escherichia coli* displays reduced uptake of several PTS carbohydrates [11]. Interestingly, L-lactate-grown *A. actinomycetemcomitans* produces extremely elevated intracellular levels of pyruvate [9], supporting a model in which elevated intracellular levels of pyruvate during catabolism of L-lactate inhibit glucose transport via reduction of the PEP:pyruvate ratio (Fig. 1).

One of the intriguing questions regarding this model is how the extremely high levels of intracellular pyruvate (approximately 50 mM) are produced during growth with L-lactate. In this study, we hypothesized that potential clues might be gained by examining the A. actinomycetemcomitans enzyme required for the first step in L-lactate catabolism, namely L-lactate oxidation to pyruvate. We show that the gene AA02749 (lctD) encodes for an NAD-independent L-lactate dehydrogenase that is critical for growth of A. actinomycetemcomitans with L-lactate. Interestingly, inhibitor studies reveal that unlike homologous enzymes, A. actinomycetemcomitans LctD maintains significant enzymatic activity, even at extremely high pyruvate levels (50 mM).

## Results

### AA02769 Is Required for Growth with L-Lactate

**Figure 2:**
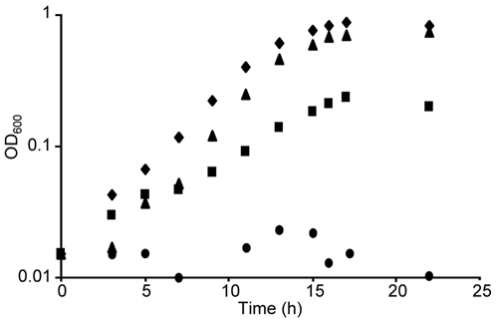
LctD is required for growth using L-lactate. Growth curves of wild-type *A. actinomycetemcomitans* and the *A. actinomycetemcomitans lctD* insertion mutant in chemically defined medium (CDM) containing 20 mM glucose or 20 mM L-lactate as the sole source of energy. Glucose-grown *A. actinomycetemcomitans* (♦), L-lactate-grown *A. actinomycetemcomitans* (▴), glucose-grown *A. actinomycetemcomitans lctD^−^* (▪), L-lactate-grown *A. actinomycetemcomitans lctD^−^* (•).

*A. actinomycetemcomitans* resides within the gingival crevice where it likely encounters levels of L-lactate ranging from 1–5 mM [5], and previous work in our lab demonstrated that this bacterium preferentially catabolizes L-lactate [9]. The first step in L-lactate catabolism is the conversion of L-lactate to pyruvate via the enzyme L-lactate dehydrogenase. Examination of the *A. actinomycetemcomitans* HK1651 genome sequence revealed two genes with high homology to lactate dehydrogenases: AA02769 (gene designations from www.oralgen.lanl.gov) putatively encodes a protein with 75% identity (E value<10^−169^ using BLASTp) to the catabolic L-lactate dehydrogenase (LctD) from *E. coli* K12 and AA02749 putatively encodes a protein with 73% identity (E value<10^−141^ using BLASTp) to the fermentative D-lactate dehydrogenase (LdhA) from *E. coli* K12. Based on the fact that LdhA homologs are primarily utilized for lactate biosynthesis in fermentative reactions [12], [13], and that *A. actinomycetemcomitans* does not grow with D-lactate as the sole carbon source (data not shown), we hypothesized that AA02769 likely encodes the enzyme required for growth on L-lactate. To test this hypothesis, the ability of an *A. actinomycetemcomitans* strain containing an insertion in AA02769 to grow with L-lactate as the sole catabolizable carbon source was assessed. Indeed, inactivation of AA02769 eliminated the ability of *A. actinomycetemcomitans* to grow with L-lactate, but not glucose (Fig. 2). Based on these results, along with those described below, we will refer to AA02769 as *lctD*.

### Over-Expression and Purification of LctD

**Figure 3:**
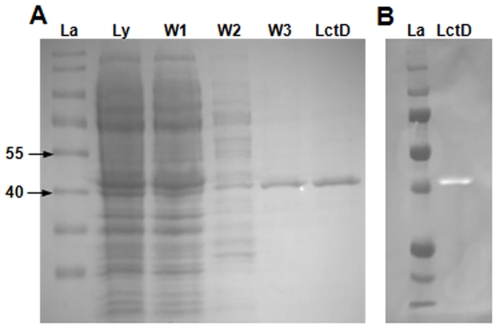
Purification of LctD-his_6_. (A) SDS-PAGE analysis of LctD-his_6_. LctD-his_6_ was purified using a HisTrap nickel column and examined by SDS-PAGE and Coomassie staining. Lane designations above the gel are: (La), molecular weight ladder; (Ly), cell lysate; (W1), buffer A flow-through; (W2), buffer B with 0.15 M imidazole flow-through; (W3), buffer B with 0.5 M imidazole flow-through; (LctD), phosphate buffer-exchanged LctD-his_6_. Numbers to the left of the SDS-PAGE gel represent size standards in kilodaltons. Phosphate buffer-exchanged LctD-his_6_ was used for enzymatic activity studies. (B) Western blot analysis of purified LctD-his_6_. Purified LctD-his_6_ was separated on a 10% SDS-PAGE gel, transferred to nitrocellulose membrane, and detected using an anti-his_6_ antibody and chemiluminescence. Image represents an overlay of a white light image and a chemiluminescent image.

*A. actinomycetemcomitans lctD* putatively encodes a 42 kDa cytoplasmic protein that is a proposed member of a family of NAD-independent L-lactate dehydrogenases that convert L-lactate to pyruvate in a unidirectional manner [14]. Due to its high homology to the catabolic L-lactate dehydrogenase from *E. coli* and the observation that inactivation of *lctD* eliminated L-lactate-dependent growth of *A. actinomycetemcomitans* (Fig. 2), we hypothesized that *A. actinomycetemcomitans* LctD catalyzes the oxidation of L-lactate to pyruvate. To test this hypothesis, *A. actinomycetemcomitans lctD* was cloned into the pET21a(+) expression vector (pSB103) to create C-terminally his_6_-tagged LctD. Affinity purification using a nickel column resulted in nearly pure LctD-his_6_ as demonstrated by a prominent band at approximately 42-kDa on a Coomassie stained gel (Fig. 3A) and a single band in a Western blot using an anti-his_6_ antibody (Fig. 3B). As a control, a purification procedure was performed using cells containing pET21a(+), which resulted in no proteins readily apparent by SDS-PAGE analysis after purification and no L-lactate dehydrogenase enzyme activity (data not shown).

In addition to C-terminally his_6_-tagged LctD, N-terminally his_6_-tagged LctD was also constructed and purified. While both purified enzymes displayed enzymatic activity, they were extremely unstable and exhibited significant loss of enzymatic activity in a variety of buffers after overnight storage at 4, −20, or −80°C; therefore all assays were carried out using freshly purified protein. Of note, protein activity from independent purifications was consistent and reproducible. LctD-his_6_ was used for enzymatic characterization as its yields were somewhat higher than those of his_6_-LctD.

### Kinetic Characterization of *A. actinomycetemcomitans* LctD

**Figure 4:**
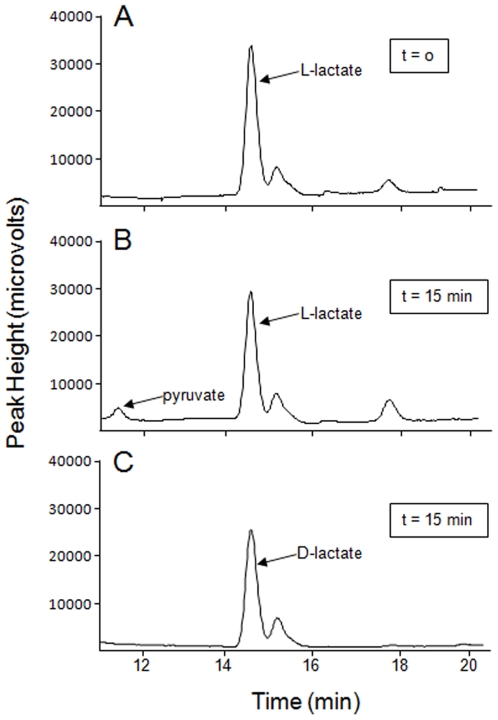
LctD-his_6_ catalyzes oxidation of L-lactate, but not D-lactate, to pyruvate. HPLC chromatogram of LctD-his_6_ in vitro enzyme reactions with (A) L-lactate as the substrate at time 0, (B) L-lactate as the substrate after 15 min, and (C) D-lactate as the substrate after 15 min. Pyruvate and D- and L-lactate were detected using a refractive index detector, and data are displayed as peak height (microvolts). Using commercially available standards, it was determined that pyruvate is detected at approximately 11.6 to 12.1 min and lactate (D and L) is detected at approximately 14.6 to 15.2 min. Representative data are shown for experiments that were performed in duplicate or triplicate.

Once purified protein was obtained, the enzymatic activity of LctD-his_6_ was assessed. The final electron acceptor of many NAD-independent enzymes is unknown, as these enzymes are likely coupled to electron transport [14]. Therefore, LctD-his_6_ activity was determined in the presence of the electron carriers 3-(4,5-dimethylthiazol-2-yl)-2,5-diphenyl-2H-tetrazolium bromide (MTT) and phenazine methosulfate (PMS) as previously described [15], [16], [17]. LctD-his_6_ catalyzed the oxidation of L-lactate to pyruvate (Fig. 4B); however, no activity was observed with the D-lactate isomer (Fig. 4C). Additionally, LctD-his_6_ was unable to catalyze the reverse reaction (pyruvate to lactate) even in the presence of the reduced substrate NADH (data not shown). These data indicate that, as expected, LctD-his_6_ is an NAD-independent L-lactate dehydrogenase that catalyzes the oxidation of L-lactate to pyruvate in a unidirectional manner.

**Figure 5:**
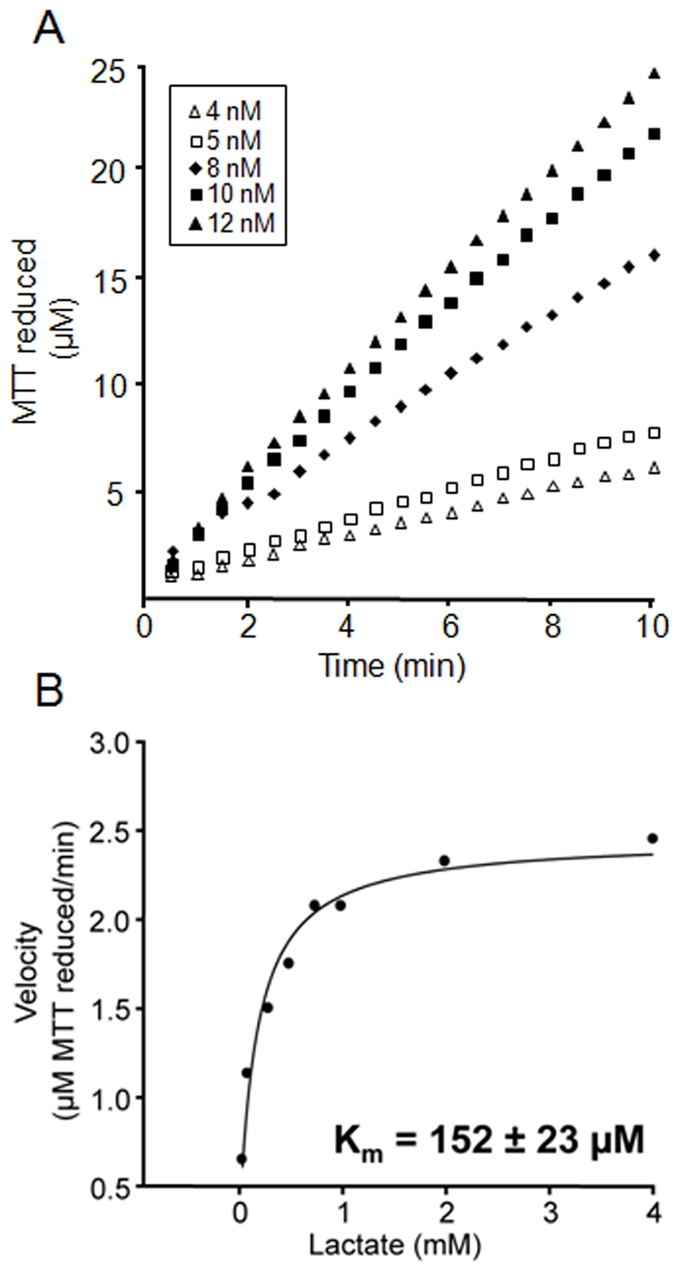
Kinetic analysis of LctD-his_6_. LctD-his_6_ was incubated with L-lactate and enzymatic activity assessed by monitoring reduction of MTT as described [15], [16], [17]. (A) Enzymatic activity was assessed over time for multiple LctD-his_6_ concentrations (4, 5, 8, 10, and 12 nM) in the presence of saturating substrate concentrations. For K_m_ calculations, 8 nM LctD-his_6_ was used. Representative data are shown. (B) The LctD-his_6_ K_m_ for lactate was calculated as 152±23 µM (average±standard deviation) by averaging values from quadruplicate experiments. Representative data are shown.

After determining that the product of LctD-his_6_ L-lactate oxidation was pyruvate, kinetic analysis was performed. For kinetic analysis, reduction of MTT (measured as the change in absorbance at 570 nm) was utilized to monitor LctD-his_6_ activity as previously described [15], [16], [17]. Activity assays were carried out in the presence of saturating substrate concentrations, and it was determined that with 8 nM enzyme, 4 mM lactate, and 60 µg/ml MTT, 240 µg/ml PMS achieved linear results for a 10 minute duration (Fig. 5A). To acquire a K_m_ value, activity assays were performed in the presence of increasing lactate concentrations, and a K_m_ value of approximately 150 µM was calculated using SigmaPlot 10.0.1 software (Fig. 5B).

### Pyruvate Is a Poor Inhibitor of LctD

**Figure 6:**
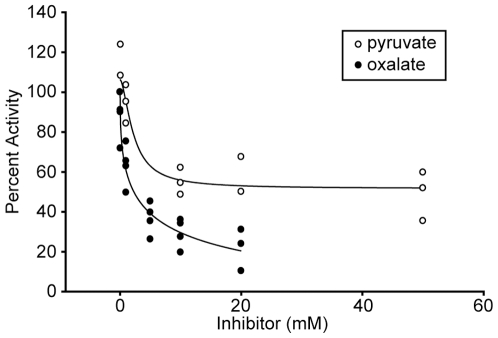
Pyruvate is a poor inhibitor of LctD-his_6_. The ability of pyruvate and oxalate to inhibit LctD-his_6_ enzymatic activity was assessed as described in Materials and Methods. 50% inhibition of LctD-his_6_ activity was observed with 10–50 mM pyruvate and 2.1 mM oxalate.

Our model for PTS substrate exclusion involves the production of high levels of intracellular pyruvate during growth with L-lactate (Fig. 1). Based on this model, we hypothesized that *A. actinomycetemcomitans* LctD-his_6_ would not be sensitive to feedback inhibition by product (pyruvate) accumulation; thus allowing intracellular accumulation of pyruvate to the high levels (50 mM) previously observed [9]. Interestingly, LctD homologs from other bacteria are often inhibited by relatively low levels (5 mM) of pyruvate [18]. To assess the impact of pyruvate on *A. actinomycetemcomitans* LctD-his_6_ activity, inhibition assays were performed in the presence of increasing concentrations of pyruvate as well as the common lactate dehydrogenase inhibitor oxalate [19], [20]. Pyruvate displayed poor inhibition of LctD-his_6_ activity, with approximately 90% activity remaining in the presence of 5 mM pyruvate and 50% activity remaining in the presence of 50 mM pyruvate (Fig. 6). As expected, oxalate was a potent inhibitor of LctD activity, with 50% inhibition observed at approximately 2 mM oxalate (Fig. 6).

## Discussion

*A. actinomycetemcomitans* is found exclusively in the mammalian oral cavity, a diverse environment where microbes likely compete for limited carbon sources including glucose, L-lactate, and fructose [4], [5]. While a large number of oral bacteria preferentially catabolize carbohydrates, previous results from our laboratory revealed that *A. actinomycetemcomitans* has evolved a preference for L-lactate despite its apparent inferiority as a carbon and energy source [9]. This unique mechanism for preferential L-lactate consumption, referred to as PTS substrate exclusion [9], may have benefits for *A. actinomycetemcomitans* as it may mitigate L-lactate-mediated acidification of the gingival crevice as well as reduce production of the antimicrobial H_2_O_2_ by oral streptococci [21]. Thus preferential consumption of L-lactate by *A. actinomycetemcomitans* may be a unique survival strategy allowing *A. actinomycetemcomitans* to compete with other members of the oral microbiota *in vivo*.

We recently proposed a model for PTS substrate exclusion in which intracellular levels of pyruvate inhibit glucose uptake in L-lactate-grown *A. actinomycetemcomitans* (Fig. 1). As this model is predicated on the accumulation of pyruvate to intracellular levels approximately 10 times higher than normally found in glucose growing bacteria [9], we hypothesized that the *A. actinomycetemcomitans* L-lactate dehydrogenase (LctD) is not feedback inhibited by high levels of pyruvate. To test this hypothesis, *A. actinomycetemcomitans* LctD was purified as a C-terminal his_6_ fusion protein and determined to be an NAD-independent L-lactate dehydrogenase that catalyzes irreversible oxidation of L-lactate to pyruvate (Fig. 4B). LctD-his_6_ displayed a K_m_ for L-lactate of approximately 150 µM, a value within the range of other characterized NAD-independent lactate dehydrogenases [14], [18], [22], including the highly homologous *E. coli* LctD enzyme (75% identity) that has a K_m_ value of 21–70 µM [22].

*A. actinomycetemcomitans* LctD-his_6_ was highly resistant to feedback inhibition by pyruvate, displaying 50% activity at 50 mM pyruvate (Fig. 6). This property is unique in that homologous enzymes in other bacteria display feedback inhibition by substantially lower levels of pyruvate. Indeed, the NAD-independent lactate dehydrogenase of *Propionibacterium pentosaceum* is inhibited by levels of pyruvate (1–5 mM) commonly observed intracellularly [18]. Our results reveal that LctD allows *A. actinomycetemcomitans* to specifically convert L-lactate to pyruvate in a unidirectional manner. Since this enzyme displays reduced inhibition by pyruvate, intracellular levels of pyruvate rise to levels known to inhibit PTS transport in other bacteria [11], [23], resulting in PTS substrate exclusion. While the PTS enzyme(s) affected by high levels of pyruvate is not known, we hypothesize that inhibition occurs at the first step of PTS transport, phosphorylation of protein EI by PEP (Fig. 1), and studies in our laboratory are currently addressing this hypothesis.

## Materials and Methods

### Bacterial Strains and Culture Conditions

*A. actinomycetemcomitans* strain VT1169 [24] and the *A. actinomycetemcomitans ldhA* mutant (referred to as the *lctD* mutant in this manuscript) [9] was grown in chemically defined Socransky's medium [25] lacking DL-mevalonic acid and hemin (referred to as Chemically Defined Medium, CDM) or tryptic soy broth with 0.5% yeast extract (TSBYE). Cultures were grown with shaking at 165 RPM at 37°C with a 10% CO_2_ atmosphere. For growth analysis, overnight TSBYE-grown cultures were washed three times with warm (37°C) CDM containing no catabolizable carbon source and diluted to an optical density at 600 nm (OD_600 nm_) of 0.015 in CDM supplemented with 20 mM L-lactate or glucose. *E. coli* DH5α and BL21(DE3) were grown in Luria broth (LB), shaking at 250 RPM at 37°C, with 75 µg/ml ampicillin for plasmid maintenance or 100 µg/ml for selection.

### Construction of N- and C-Terminally Tagged LctD

The *lctD* gene from *A. actinomycetemcomitans* strain 1169 was amplified by PCR using primers lctDN-for (5′-GGAATTCCATATGATTATTTCGTCCGCTAACG-3′) and lctDN-rev (5′- CCGCTCGAGGCGTATATAAAATACGCCGTTTG-3′) or lctDN-for and lctDC-rev (5′- CTCGAGCTTACTTAAATCTACTAATGC-3′) to create the N-terminally or C-terminally his_6_-tagged constructs respectively. Forward primers contained an NdeI site and reverse primers contained an XhoI site. Products were digested with NdeI and XhoI and ligated into the NdeI/XhoI-digested pET15b expression vector (Novagen) for the his_6_ N-terminal tag or the NdeI/XhoI-digested pET21a(+) expression vector (Novagen) for the his_6_ C-terminal tag. The resultant plasmids were transformed into *E. coli* DH5α and sequenced using T7 promoter and SP6 terminator primers. Resulting plasmids were named pSB201 (his_6_ N-terminal tag) and pSB203 (his_6_ C-terminal tag). Plasmids were transformed into the expression strain *E. coli* BL21(DE3) for expression and purification.

### Protein Purification

*E. coli* BL21(DE3) carrying pSB201 or pSB203 were grown in TB (EMD) containing 75 µg/ml ampicillin to an OD_600 nm_ = 0.6, and induced overnight at 16°C with 100 µM IPTG in the presence of 100 µM riboflavin (Sigma). Induced cells were harvested by centrifugation for 15 minutes at 6100×g in a Beckman Coulter Avanti J–E centrifuge at 4°C. The pellet was resuspended in 3 ml buffer A (25 mM phosphate buffer, 0.5 M NaCl, 20 mM imidazole, 10 µM flavin adenine dinucleotide (FAD), pH 7.07) containing one-half tablet complete Mini protease inhibitor cocktail (Roche), 25 U Benzonase Nuclease (Novagen) and 10 µM FAD (Alfa Aesar). The cells were passed three times through a French press (American Instruments Company) at 20,000 pounds/square inch. The resulting lysate was centrifuged at 60,000×g in a Beckman-Coulter OptimaL 100 K Ultracentrifuge for one hour at 4°C to remove cellular debris and insoluble protein. The lysate was then applied to a HisTrap HP column (GE Healthcare). The column was washed with 3 ml cold buffer B (25 mM phosphate buffer, 0.5 M NaCl, 10 µM FAD, pH 7.07) containing 0.15 M imidazole, followed by elution with cold buffer B containing 0.5 M imidazole. The eluted protein was concentrated, and a buffer exchange was performed with 25 mM phosphate buffer containing 10 µM FAD. Samples were separated on a 10% SDS-PAGE gel and stained with Coomassie Brilliant Blue (Pierce). Bradford analysis to quantify protein was performed with the Bio-Rad Protein Assay as outlined by the manufacturer. Western blot analysis was performed as outlined [26] using a commercially available anti-his_6_ antibody (Sigma) and a stabilized goat anti-mouse HRP-conjugated secondary antibody (Pierce). Chemiluminescent detection was performed using the SuperSignal West Dura Extended Duration Substrate as outlined by the manufacturer (Thermo). Blots were imaged with a G-box gel documentation system (Syngene). As a control, a purification procedure was carried out as described above with BL21(DE3) cells containing the parent plasmid pET21a(+).

### Characterization of LctD-his_6_ Enzymatic Activity

Activity assays were performed with phenazine methosulfate (PMS) (Acros Organics) and 3-(4,5-dimethylthiazol-2-yl)-2,5-diphenyl-2H-tetrazolium bromide (MTT) (Calbiochem) as previously described [15], [16], [17]. To initially examine enzymatic activity of LctD-his_6_, reactions (1.5 ml) were carried out in 0.08 M Tris buffer containing 120 µg/ml PMS, 60 µg/ml MTT, 2 mM substrate (L-lactate, D-lactate, or pyruvate), and 100 nM LctD-his_6_. Reactions containing pyruvate as the substrate also contained 1 mM NADH. Half of the reaction volume was removed at time zero, heated at 65°C for 5 minutes, chilled on ice, and stored at −80°C overnight. The remaining half of the reaction was processed in the same manner at 15 minutes, and all samples were filtered through a 0.2 µm Nanosep centrifugal device (Pall). Product analysis was carried out on a Varian HPLC using a Varian Metacarb 87H 300×6.5 mm column at 35°C. Samples were eluted with 0.025 N H_2_SO_4_ isocratic elution buffer with a flow rate of 0.5 ml/minute. A Varian refractive index (RI) detector at 35°C was used for product detection with commercially available L-lactate, D-lactate, and pyruvate as standards.

For kinetic analysis, assays were performed monitoring a PMS-coupled reduction of MTT as previously described [15], [16], [17]. The assay was performed in 0.08 M Tris-HCl (pH 8.6) containing 60 µg/ml MTT, 240 µg/ml PMS, and a range of purified enzyme. Absorbance changes in MTT were measured at 570 nm over 10 minutes. Absorbance values obtained from enzyme containing reactions were adjusted by subtracting background absorbance values from a reaction containing no enzyme. Calculations were performed using an extinction coefficient of 17 mM^−1^ cm^−1^ [27]. For inhibition studies, the activity assays described above were performed in the presence of increasing concentrations (0.05 to 50 mM) of pyruvate (Alfa Aesar) or oxalate (Fisher).
